# 3D-printed custom-made short stem with porous structure for fixation of massive endoprosthesis in joint‐preserving reconstruction after tumor resection

**DOI:** 10.1186/s13018-023-03954-8

**Published:** 2023-06-29

**Authors:** Zhuangzhuang Li, Minxun Lu, Yuqi Zhang, Taojun Gong, Li Min, Yong Zhou, Yi Luo, Chongqi Tu

**Affiliations:** 1 Department of Orthopedics, Orthopaedic Research Institute, West China Hospital, Sichuan University, No. 37 Guoxue Road, Chengdu, 610041 Sichuan People’s Republic of China; 2 Model Worker and Craftsman Talent Innovation Workshop of Sichuan Province, West China Hospital, Sichuan University, Chengdu, People’s Republic of China

**Keywords:** 3D-printed, Reconstruction, Bone defect, Short-segment fixation

## Abstract

**Background:**

Large malignant bone tumors and revision limb salvage procedures often result in massive bone loss, leaving a short residual bone segment that cannot accommodate a standard stem for endoprosthesis fixation. Three-dimensional-printed (3DP) short stem with porous structure seems to be an alternative for short-segment fixation. This retrospective study aims to evaluate surgical outcomes, radiographical results, limb functions, and complications of using 3DP porous short stems in massive endoprosthesis replacement.

**Methods:**

Between July 2018 to February 2021, 12 patients with massive bone loss undergoing reconstruction with custom-made, short-stemmed massive endoprostheses were identified. Endoprosthesis replacement involved the proximal femur (*n* = 4), distal femur (*n* = 1), proximal humerus (*n* = 4), distal humerus (*n* = 1), and proximal radius (*n* = 2).

**Results:**

The mean percentage of resected bone was 72.4% of the whole length of the bone, ranging from 58.4 to 88.5%. The mean length of 3DP porous short stems was 6.3 cm. The median follow-up was 38 months (range, 22–58 months). The mean MSTS score was 89%, ranging from 77% to 93%. Radiographical assessment results showed bone in-growth to the porous structure in 11 patients, and the implants were well osseointegrated. Breakage of the 3DP porous short stem occurred in one patient intraoperatively. The patient developed aseptic loosening (Type 2) four-month after surgery and underwent revision with a plate applied to assist fixation. The implant survivorship was 91.7% at 2 years. No other complications were detected, such as soft-tissue failures, structural failures, infection, or tumor progression.

**Conclusions:**

3DP custom-made short stem with porous structure is a viable method for fixation of the massive endoprosthesis in the short segment after tumor resection, with satisfactory limb function, great endoprosthetic stability, and low complication rates.

**Supplementary Information:**

The online version contains supplementary material available at 10.1186/s13018-023-03954-8

## Background

Limb salvage surgery has been the standard treatment for bone and soft-tissue tumors in extremities due to advances in imaging modalities, neoadjuvant chemotherapy, and surgical techniques [1–3]. Compared with biological reconstruction, endoprosthesis replacement following tumor resection brings many advantages, including immediate stability, rapid rehabilitation, and early weight bearing [4, 5]. In the last decades, modular stemmed endoprosthesis has become a widely used, preferred modality for the reconstruction of osteoarticular defects of the upper and lower extremities [6, 7]. However, large malignant bone tumors and revision limb salvage procedures often result in massive bone loss, leaving a short residual bone segment that cannot accommodate a standard intramedullary stem [8].

In this situation, a short-stemmed endoprosthesis is a common choice to avoid total endoprosthesis replacement which impairs function significantly because of sacrificing two native joints [9]. Nevertheless, short-stemmed endoprostheses conceivably have an increased risk of aseptic loosening [8]. Nowadays, several techniques have been developed especially to improve the fixation efficacy in the short segment, such as compress osseointegration stems [10, 11], short stems with cross-pin [12, 13], or extra-cortical plate [8], and telescope allograft augment technique [14]. Using these techniques, a 9–22% failure rate due to aseptic loosening or structural failure at short- to long-term follow-up has been demonstrated.

Recently, three-dimensional-printed (3DP) endoprosthesis has become a powerful tool for complex reconstruction in extremities, improving limb and joint salvage rates [15]. Moreover, a 3DP short stem with porous structure seems to be an alternative for short-segment fixation and has been reported that creates a stable endoprosthesis fixation in intercalary reconstruction [16–18]. However, the clinical efficacy and outcomes of using this technique in osteoarticular reconstruction for fixation of the massive endoprosthesis remain unclear. Previously, resection of an extensive length of bone has been shown associated with implant failure, and the greater the percentage of bone resected, the greater the probability of failure [19]. Therefore, it is necessary and interesting to examine whether 3DP porous short stem is an alternative for short-segment fixation of the massive endoprosthesis.

This retrospective study aims to evaluate surgical outcomes, radiographical assessments, limb functions, and complications of using 3D-printed porous short stems in massive endoprosthesis replacement.

## Methods

### Patients

Institutional review board approval was obtained for this retrospective study. Twelve patients with massive bone loss underwent reconstruction with 3DP custom-made, short-stemmed massive endoprostheses between July 2018 to February 2021 were identified. A short stem was defined as being < 100 mm in length. A total of nine primary reconstructions following primary tumor resection and three revision procedures were performed. According to the anatomical site, endoprosthesis replacement involved the proximal femur (*n* = 4), distal femur (*n* = 1), proximal humerus (*n* = 4), distal humerus (*n* = 1), and proximal radius (*n* = 2). There were five females and seven males, with a mean age at the time of diagnosis of 35 years. Preoperatively, all patients underwent detailed radiography examinations, including X-ray, computed tomography (CT), and magnetic resonance imaging (MRI) of the affected limb. Details of each patient’s diagnosis and clinical characteristics were collected and are shown in Table 1.

**Table 1:** Demographics, clinical data, and follow-up results of 12 patients

Patients	Sex	Age, year	Site	Diagnosis	Resection length, cm/percentage, %	Stem length, cm	Assisted fixation	Follow-up, months	MSTS, %	Complications	Patients status
1	M	67	Proximal femur (Right)	CS	31/88.5	3.0	Screw	58	90	NA	NED
2	F	23	Proximal femur (Left)	OS	28.6/73.5	6,8	Screws	46	93	NA	NED
3	M	51	Proximal femur (Right)	CS	30.6/71.2	7.7	Screws	53	93	NA	NWD
4	F	20	Proximal femur (Left)	OS	27.2/75.2	6.3	Screws	39	90	NA	NED
5	F	43	Distal femur (Right)	Revision	29.0/78.4	8.6	Screw	34	93	Periprosthetic fracture	NED
6	M	36	Proximal humerus (Left)	OS	12.4/54.8	70	NA	24	90	NA	NED
7	M	61	Proximal humerus (Left)	Revision	15.07/63.4	6.8	Plate; screws	50	93	NA	NED
8	M	15	Proximal humerus (Left)	OS	20.2/61.3	8.2	Screws	39	87	NA	NED
9	M	49	Proximal humerus (Left)	Revision	17.1/58.4	9.6	NA	37	87	NA	NED
10	F	17	Distal humerus (Right)	OS	23.7/84	3.7	Screws	22	90	NA	NWD
11	F	11	Proximal radius (Left)	ES	12.5/76	3.0	Plate; screws	31	77	Stem breakage, Dislocation	NED
12	M	28	Proximal radius (Right)	SS	22.2/84	3.0	NA	29	87	NA	NWD

*CS* chondrosarcoma, *OS* osteosarcoma, *ES* Ewing’s sarcoma, *SS* synovial sarcoma, *NA* none, *NED* no evidence of disease

### Endoprosthesis properties

**Fig. 1:**
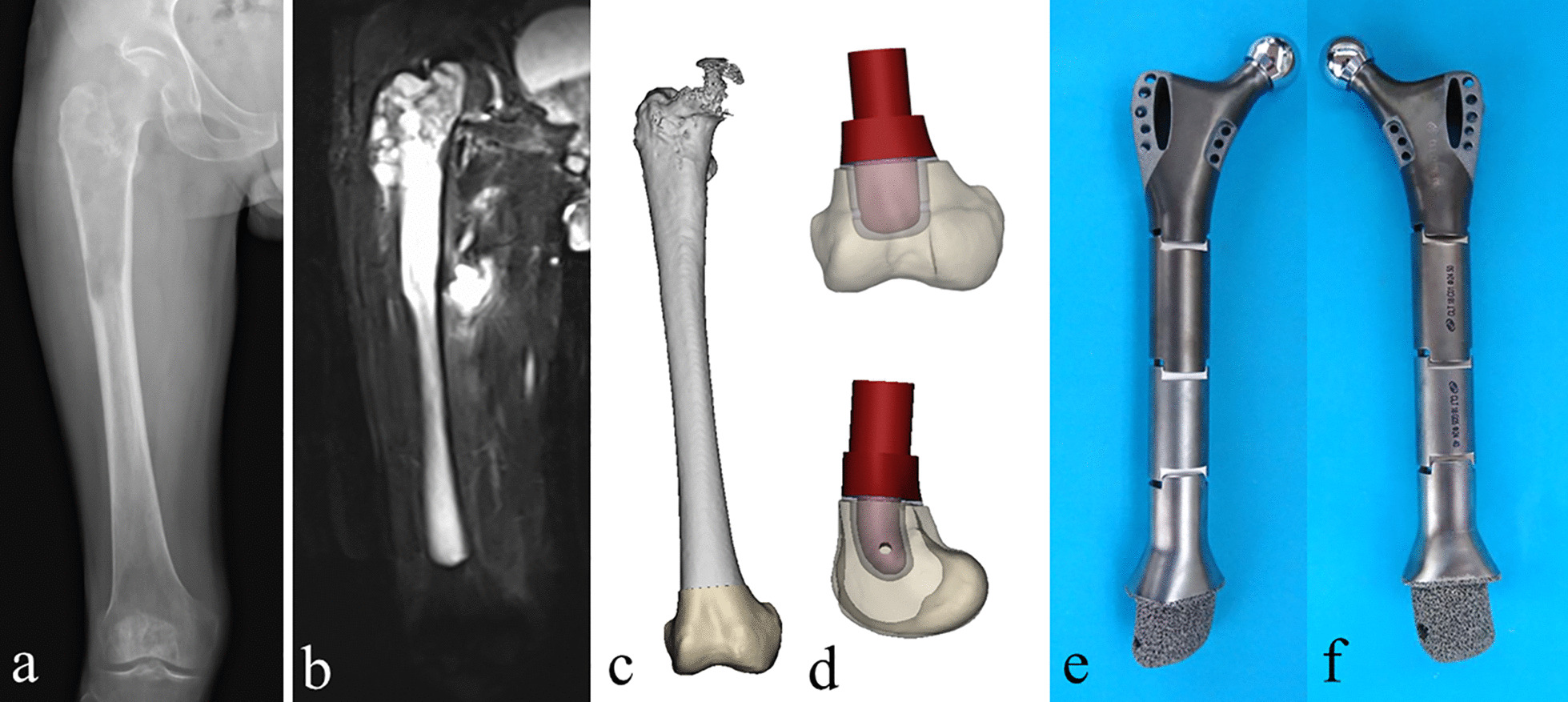
Preoperative X-ray (**a**) and MRI image (**b**) of a man aged 67 years with chondrosarcoma involving the proximal femur; **c** simulating tumor resection; **d** designing porous short stem; **e**, **f** photographs of the custom-made porous short stem assembled with a modular endoprosthesis

All endoprostheses were custom-made for each patient, and all short porous stems were designed by our clinical team. Firstly, CT scan data (DICOM format) were collected and imported into Mimics software for reconstructing 3D models (Fig. 1). After that, osteotomy was simulated based on the safe excision margin determined by pre-operative X-ray, CT, and MRI. The short stem was designed by imitating the shape of the remaining medullary cavity, which was described in detail in our previous articles [16, 20]. A total of three types of short stems were designed for fixation of the endoprosthesis in the short residual bone segments: diaphysis curved stem in the distal femur segment, intra-neck curved stem in the proximal femur segment, and straight stem in the proximal humerus, distal humerus, and distal radius segment. After that, the stem (STL format) was separated into two parts: an internal solid body and an external porous structure layer (2.5 mm in the femur, 1.5 mm in the humerus, and 1 mm in the radius). Lastly, modular endoprostheses were prepared for the proximal femur replacement, the distal femur replacement, and the proximal humerus replacement. And bionic hemi-elbow implants were prepared for the distal humerus and proximal radius replacement (Additional files 1).

**Fig. 2:**
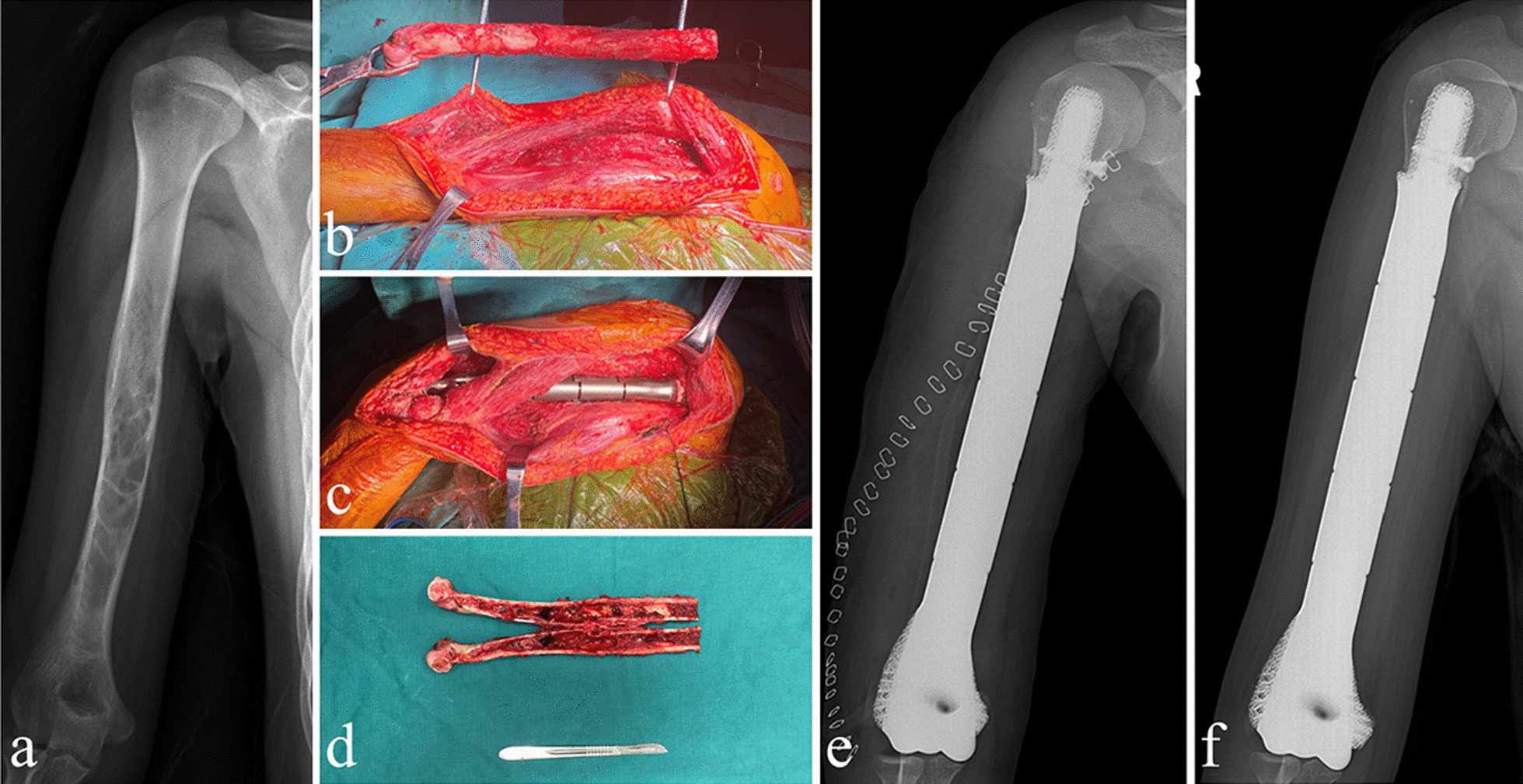
A woman aged 17 years with osteosarcoma underwent reconstruction with a 3DP custom-made short-stemmed endoprosthesis. **a** Preoperative X-ray; **b**–**d** intraoperative pictures; X-ray after surgery immediately and (**e**) and 12 months (**f**) after surgery

All the implants were fabricated by Chunli Co., Ltd. (Tongzhou, Beijing, China). The short stems with the porous structure and bionic hemi-elbow implants were fabricated using the electron beam melting technique (ARCAM Q10plus). And the modular endoprostheses were manufactured through the forging method, which can be assembled with the 3DP porous short stem.

### Surgical procedure and postoperative management

All the surgeries were performed by the same senior surgeon (Fig. 2). After general anesthesia, the patient's position and surgical approach were selected based on the location of the tumor to obtain adequate tumor exposure. Careful dissection of the soft tissue, as well as identification and protection of related major neurovascular structures, were performed. And then, osteotomies were undertaken precisely according to the preoperative design. Next, the medullary cavity was reamed, and the porous short stem was inserted into the prepared medullary cavity. The insertion of transverse screws was determined by the stability evaluation intraoperatively. At last, soft-tissue coverage of the endoprosthesis was performed.

Postoperatively, the operative lower limb was kept non-weight bearing with a splint or brace for 4–6 weeks after surgery. Thereafter, patients were encouraged to gradually increase weight bearing on the affected limb, while for the upper limbs, the operative limb was protected with a brace for 3–4 weeks after surgery. After that, the patients were allowed to have motion as tolerated.

### Follow-up and evaluation

**Fig. 3:**
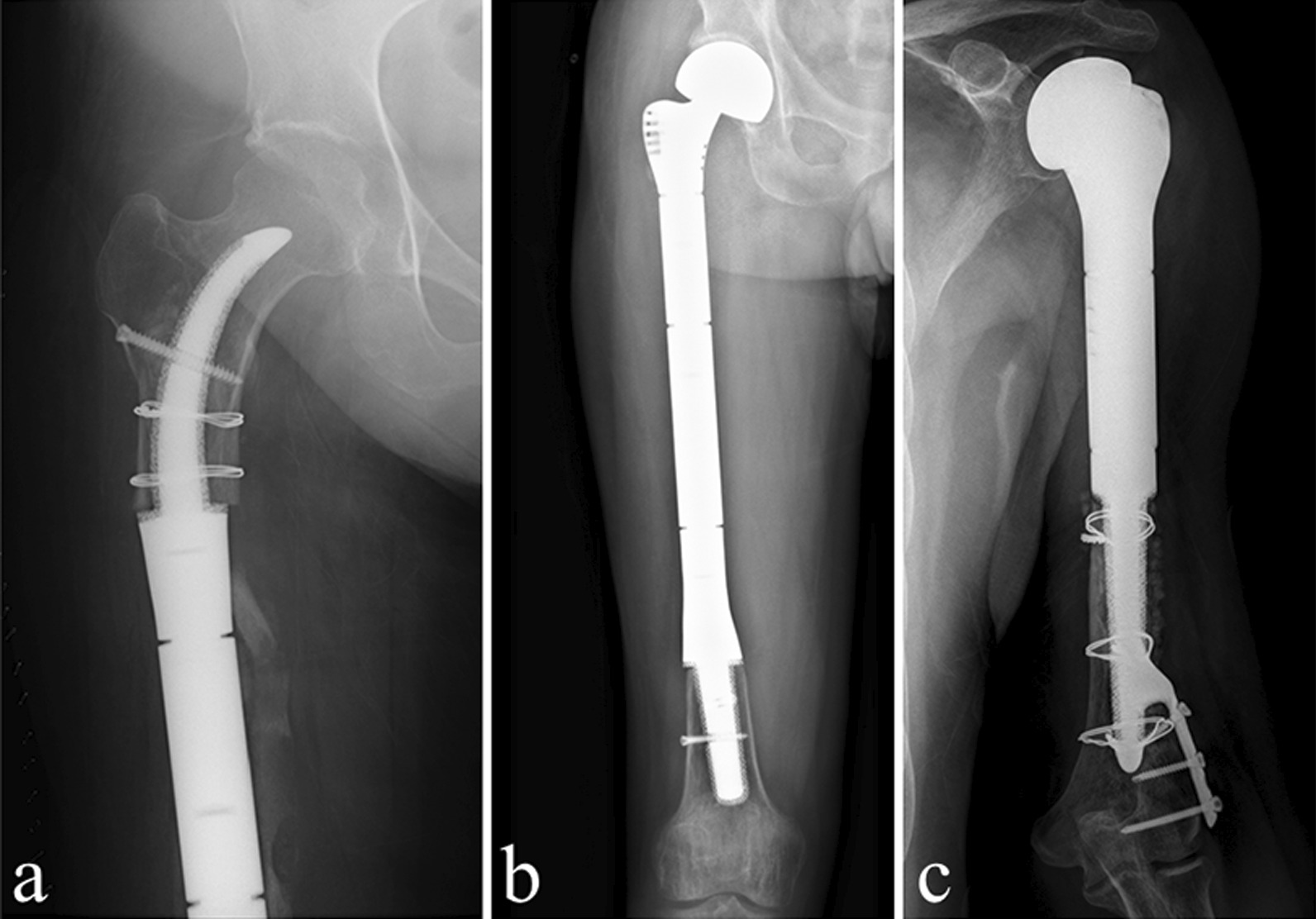
**a** Intra-neck curved porous short stem in the proximal femur segment; **b** diaphysis curved short stem in the distal femur segment; **c** porous short stem with an extra-cortical plate in the distal humerus segment

All patients were followed up once a month for the first 3 months and every 3 months thereafter. At each follow-up, the patients underwent detailed physical examinations. X-ray was performed to evaluate the implant status, regularly (Fig. 3). In addition, the T-SMART was used to evaluate the bone in-growth to the porous structure. The surgical outcomes, including intraoperative complications, operative time, and blood loss, were collected from the operation records. The limb functions were evaluated according to the Musculoskeletal Tumor Society (MSTS) scoring system. Complications were categorized according to the Henderson classification: soft-tissue failure (Type 1), aseptic loosening (Type 2), structural failure (Type 3), infection (Type 4), and tumor progression (Type 5) [21].

### Statistical analysis

Descriptive statistics including medians, means, and percentages were conducted. Kaplan–Meier survival analysis was used to analyze implant survivorship, which was defined as the time from primary endoprosthesis replacement to revision surgery due to any reason.

## Results

The mean resection length was 22.5 cm (12.5–31 cm). The mean percentage of resected bone was 72.4% of the whole length of the bone, ranging from 58.4 to 88.5%. The mean length of the 3DP porous short stem was 6.3 cm. The median follow-up was 38.5 months (range, 22–58 months). At the latest follow-up, all 12 patients were disease-free, without local tumor recurrence or distant metastasis.

### Surgical outcomes

Breakage of the 3DP porous short stem occurred in one patient intraoperatively, leaving only the proximal part of the stem preserved for fixation. For another 11 patients, the endoprosthesis was successfully implanted according to the postoperative planning, without any intraoperative nerve or vessel injury occurring. While there was one periprosthetic fracture intraoperatively, and a wire was applied to assist fixation. The mean operative time was 168 min, and the mean intraoperative blood loss was 430 ml.

### Radiological and functional outcomes

**Fig. 4:**
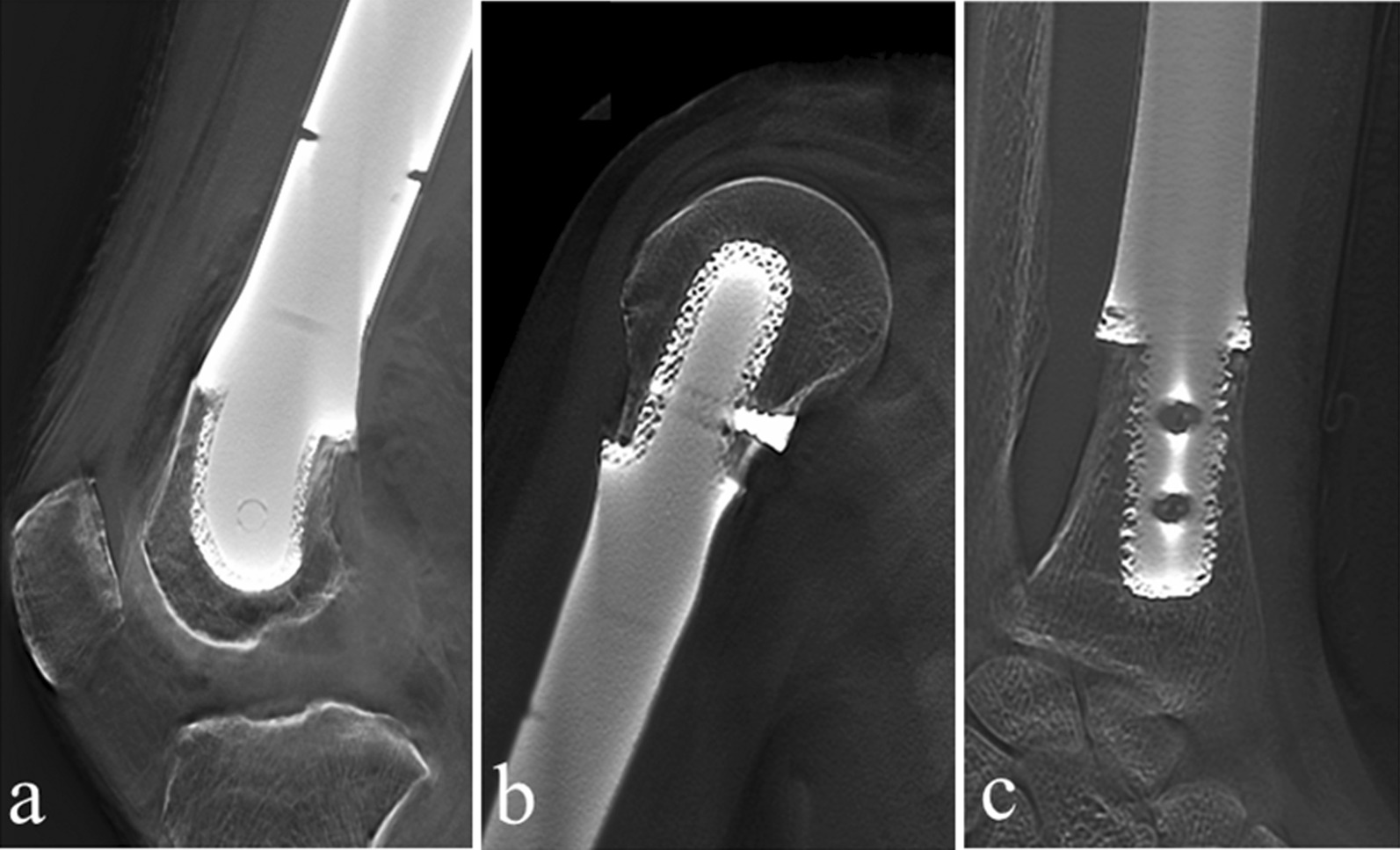
T-SMART images showed that implants were well osseointegrated in short segments following resection of bone > 80% of the whole length of the bone. **a** In the distal femur segment; **b** in the proximal humerus segment; and **c** in the distal radius segment

Bone in-growth to the porous structure was seen in all 11 patients with successful implantation (Fig. 4). And the radiographic results revealed good interfaces in the 11 3DP porous stems. After surgery, all patients experienced satisfactory limb function, and the mean MSTS score was 89%, ranging from 77% to 93%.

### Complications

**Fig. 5:**
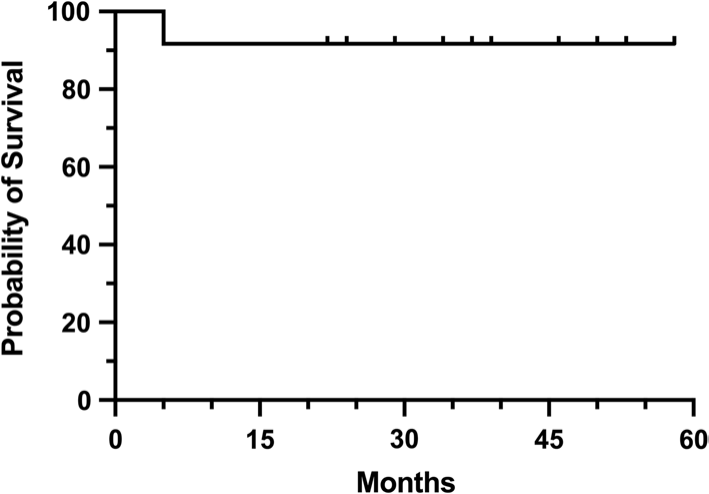
Graph showing implant survivorship of 12 patients

The patient with intraoperative breakage of the stem developed aseptic loosening (Type 2) four-month after the surgery (Additional file 2). Revision surgery was performed with a plate applied to assist fixation. The endoprosthesis condition was good at the last follow-up. In the remaining 11 patients, no other complications were detected, such as soft-tissue failures, structural failures, infection, or tumor progression. The implant survivorship was 91.7% at 2 years (Fig. 5).

## Discussion

Limb-sparing surgery for patients with extensive bone loss, which often results in a short residual bone segment, remains a great challenge for surgeons. An alternative is using 3DP porous short stem for fixation of the massive endoprosthesis in the short segment. This approach aims to promote osseointegration and permanent biologic fixation by providing a porous interface. This paper retrospectively analyzed the clinical results of 3DP porous short stem in massive endoprosthesis replacement. Exciting results were observed in our series, with satisfactory limb function, great endoprosthetic stability, and low complication rates.

In all 12 patients, following sacrificing one joint inevitably, successful preservation of another native joint was achieved, and the bone defects were successfully reconstructed with short-stemmed endoprostheses. As function outcomes, MSTS scores at the last follow-up averaged 89%, comparable with the results of modular standard length stemmed endoprosthesis in other publications [22, 23]. Other than short-stemmed endoprosthesis, total femur/humerus replacement is another common option for the treatment of large malignant bone tumors of the femur/humerus. In a study by Sevelda et al. [24], 34 patients undergoing conventional and 10 patients undergoing expandable total femur replacements were followed up for a mean of 57 months and 172 months, respectively. The mean MSTS score was 70% (27–97%) of total femur replacement and 88% (60–97%) of expandable total femur replacement, respectively. Schneider et al. [25] reported the functional outcome of total humeral replacement after tumor resection; the median MSTS score in 9 of 13 surviving patients after a median follow-up of 75 months was 87% (67–92%). Compared with total femur/humerus replacement, 3DP short-stemmed endoprosthesis avoided the sacrifice of two native joints, resulting in better limb function.

In the present study, the implants were well osseointegrated in all 11 patients with successful implantation of 3DP porous short stems. And the radiographic assessment results revealed good interfaces in the 11 3DP porous stems. When fixation of the endoprosthesis in a short segment, a short stem reduces the bone/cement interface or bone in-growth interface, and therefore the endoprosthetic stability is reduced. Previously, cross-pin [10, 11] and extra-cortical plate [8] were frequently selected to assist the stability of the short stem. Despite the initial stability being relatively easy to secure with the assistance of these two techniques, concerns regarding the long-term survival of endoprostheses remain due to their high failure rates. Additionally, fixation with an extra-cortical plate requires adequate cortex exposure, which might imperil the attachment of non-osseous tissue around the joint and therefore impair joint stability. In addition, the telescope allograft technique is developed to augment the length of the native bone when residual bone stock is insufficient for a standard stem, with the advantage of lengthening the bone stock [14]. But the use of allograft inevitably involves the complications related to poor integration with host bone, such as a nonunion and delayed union. In our study, porous short stems were custom-made to match the shape of the remaining medullary cavity. The stem with an ellipse cross section, curved shape, and transverse screws enabled primary fixation. And the plate was only selected to assist initial stability in patients undergoing revision, in which the medullary cavity was incomplete after primary replacement failure. The stem was equipped with a porous structure rather than a coating surface, to facilitate osseointegration. Therefore, custom-made porous short stems created great endoprosthetic stability in our series.

Aseptic loosening remains a common cause of megaprosthetic reconstruction failure in the current generation of implants [26]. Further study has shown that the presence of a radiolucent area of more than 20% without cortical expansion remodeling is an important risk main of loosening due to the contact area of the bone-endoprosthesis decreases [27]. Similarly, short residual bone stock after extensive tumor resection restricts the stem length, and therefore also reduces the contact area of the bone-endoprosthesis. It has been suggested that a greater than 50% of the resection length to the whole length of bone might lead to a higher rate of loosening [9]. A study by Guo et al. also found that a resection length of greater than 14 cm independently predicted the failure of cemented endoprostheses [28]. In the present study, 3DP porous short-stemmed endoprosthesis seems to be not insensitive to resection length. The percentage of resection of bone was > 50% of the length of the bone in all patients, but ranged from 58.4 to 88.5% of the whole length of the bone. Except for one case with 3DP short stem breakage intraoperatively, no aseptic loosening was observed with a median follow-up of 38 months in 11 patients with successful implantation of the stem. Anatomically, a short residual metaphyseal segment is reverse-funnel-shaped, with a larger diameter in shorter the bone segment. This means that the stem is limited in length in the shorter bone segment, while the diameter of the stem can increase. Therefore, the porous interface of the 3DP short stem for osteointegration can be secure in a shorter stem with a larger diameter.

In this present study, only one complication needs further revision. Indeed, that was a design mistake, the transverse screws reducing the internal solid body of the stem and impairing the strength. With regard to the design of the porous stem for endoprosthesis fixation in a thin medullary cavity, consideration should be given to using the transversal screws carefully. In addition, no other complications were detected. The implant survivorship was 91.7% at 2 years (Additional file 1).

Certain limitations of this study should be noted. First, this is a retrospective study with no comparison group or control group. Secondly, the number of patients included in this study is relatively small. Third, the time of follow-up was relatively short, and the clinical outcome needs to be further investigated with long-term follow-up (Additional file 2).

## Conclusion

3DP custom-made short stem with porous structure is a viable method for fixation of the massive endoprosthesis in the short segment after tumor resection, with satisfactory limb function, great endoprosthetic stability, and low complication rates.

## Supplementary Information

**Additional file 1.** Video showing bionic implant design of proximal radius.

**Additional file 2.** A child aged 11 years with breakage of the porous short stem intraoperatively. (a) X-ray after the surgery showing only the proximal part of the stem preserved and inserted the residual segment; (b) X-ray four months after the surgery showing aseptic loosening; (c) revision was performed with a plate applied to assist fixation.

## Data Availability

The datasets used during the current study are available from the corresponding author on reasonable request.
