# Supplementary figures and images for: 3D-printed custom-made short stem with porous structure for fixation of massive endoprosthesis in joint‐preserving reconstruction after tumor resection
Source: J Orthop Surg Res. 2023 Jun 29;18:468. doi: 10.1186/s13018-023-03954-8 (PMC10308689; doi:10.1186/s13018-023-03954-8)

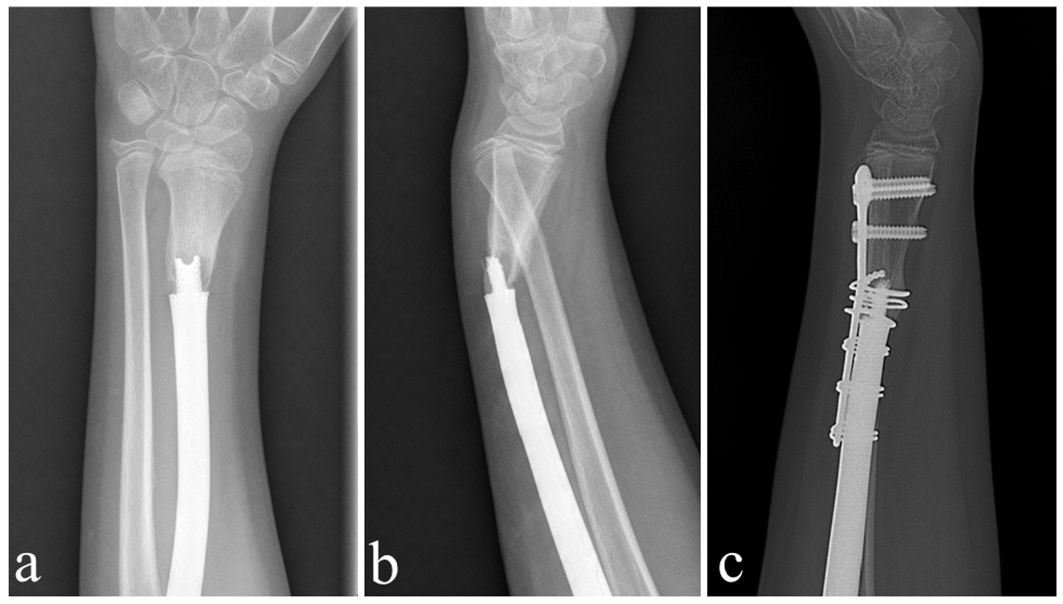

Supplement: Supplementary file 2 — Additional file 2. A child aged 11 years with breakage of the porous short stem intraoperatively. (a) X-ray after the surgery showing only the proximal part of the stem preserved and inserted the residual segment; (b) X-ray four months after the surgery showing aseptic loosening; (c) revision was performed with a plate applied to assist fixation. [file 13018_2023_3954_MOESM2_ESM.tif]
